# Vaginal and Uterine Discharges

**Published:** 1851-01

**Authors:** Thomas Weeden Cooke

**Affiliations:** Medical Officer for the Diseases of Children at the Royal Free Hospital


### ARTICLE III.

*Oak Bark and Alum Injections in Vaginal and Uterine Discharges.* By THOMAS WEEDEN COOKE, Esq., Medical Officer for the Diseases of Children at the Royal Free Hospital.

Some months since a lady placed herself under my care, for a slight prolapsus of the uterus, induced, as she supposed, by violent exertion in lifting her husband (a large man suffering from gout) into bed. Besides the prolapsus, there was also a polypus, protruding from and distending the mouth of the uterus, and there had been, previous to my seeing her, considerable leucorrhœa. Upon digital examination, I was surprised to find, considering she had had children, and was past the meridian of life, so contracted an inlet that the index finger was, with great pain and difficulty, passed into the vagina; there was also a roughness and dryness of the mucous lining, and very considerable heat; the bowels were very much confined; there was ardor urinæ, whilst the whole system was affected with a symtomatic fever, suggestive of local inflammation; hot skin; thirst; dry tongue; small, quick pulse, &c. Such violent symptoms appeared strange and unaccountable, until the history of the case disclosed the cause of all this distress. For some weeks she had been injecting decoction of oak-bark with alum, and had not taken the precaution to wash out the congealed albumen of the leucorrhœal discharge. The consequence was, this leathery compound collected upon the walls of the vagina, and os and cervix uteri, contracting the external orifice, and inducing all the inflamatory symptoms I have described. I was enabled to remove a small portion only of the offending matter with my finger; and on the next day Mr. Locock saw her with me, and suggested another attempt, but the pain produced was so great that it was impossible to succeed entirely. By warm water injections, however, some lubricating ointment, and saline aperients, the vagina was cleared in about a week, and all the urgent inflammatory symptoms subsided. A case in every respect similar has just occurred to me, although the symptoms were less severe, and the same means of relief were resorted to with beneficial result. Whilst in consultation with Dr. Locock on the former case, he kindly gave me the history of a still more interesting one, which occurred in his practice some years ago, where a large sausage-like portion of hardened albumen proceeded from the upper part of the vagina, and had been mistaken for a hernia of the bowel. He, however, broke off the supposed hernia, and removed it, much to the wonderment of the attendants. I trust Dr. Locock will pardon me for mentioning this case without his consent, as indeed, I am sure he will. if it should be considered likely to prove a serviceable warning, or rather reminder; for, of course, we all know, although we may not always think to warn our patients of it, that these astringent injections do solidify the albuminous discharges females are so obnoxious to; and may produce the evil consequences these cases represent. For myself, I always request the patient to syringe with water three or four times, both before and after using the astringent. —*London Lancet.*

